# Rapid Cycle Deliberate Practice: Application to Adult Advanced Life Support

**DOI:** 10.15766/mep_2374-8265.11269

**Published:** 2022-08-23

**Authors:** Erin Blanchard, David Booker, Dawn Taylor Peterson, Tekuila Carter

**Affiliations:** 1 Assistant Professor, Department of Health Services Administration, and Associate Director, Anesthesiology and Perioperative Medicine Simulation Program, University of Alabama at Birmingham; 2 Second-Year Resident, Department of Emergency Medicine, Baylor Scott and White Medical Center; 3 Associate Professor, Departments of Medical Education and Health Services Administration, and Director, Office of Interprofessional Simulation for Innovative Clinical Practice, University of Alabama at Birmingham; 4 Associate Professor and Residency Director, Department of Anesthesiology, University of Alabama at Birmingham

**Keywords:** Simulation, Rapid Cycle Deliberate Practice, Advanced Cardiac Life Support, Anesthesiology, Cardiovascular Medicine, Emergency Medicine

## Abstract

**Introduction:**

This curriculum includes three in-person simulation cases for Advanced Cardiac Life Support (ACLS) training using the rapid cycle deliberate practice (RCDP) technique. RCDP is a model for simulation-based medical education (SBME) that provides frequent feedback and opportunities to practice techniques until learning is cemented. The intent of these cases was to improve teamwork and communication, role designation, defibrillator operation, leadership, and clinical treatment of cardiac emergencies.

**Methods:**

Each case provided an ACLS scenario for an adult patient in the postanesthesia care unit setting. The curriculum required high-fidelity mannequins and instructors trained to provide SBME through RCDP. Learners worked in teams and were expected to perform appropriate steps per the ACLS algorithm, with facilitators pausing learners and providing expert feedback and opportunities for deliberate practice throughout.

**Results:**

Eighty-four postgraduate year 2 anesthesiology residents participated in the simulation curriculum over eight course offerings. Facilitators noted improved communication and teamwork among participants, as well as more accurate and effective defibrillator use. Feedback from learners was positive and indicated that they believed the experience would improve their clinical performance.

**Discussion:**

This curriculum provides instruction on using the RCDP variant of SBME to prepare health care providers to deliver effective care in situations necessitating ACLS. Because RCDP allows for repeated iterations of the same skill, knowledge can be cemented and muscle memory created. Given the positive feedback, we believe the curriculum can provide an effective framework for ACLS reinforcement through RCDP implementation across multiple types of learners and institutions.

## Educational Objectives

By the end of this activity, learners will be able to:

1. Demonstrate effective teamwork and communication.
2. Appropriately designate roles.
3. Effectively operate a defibrillator in manual mode.
4. Successfully lead a team during a cardiac emergency.
5. Accurately apply Advanced Cardiac Life Support algorithms to patient care.

## Introduction

Simulation-based medical education (SBME) is an effective instructional strategy for incorporating deliberate practice and mastery learning^1^ and has been shown to be an effective method of improving medical practice that has advantages over traditional forms of instruction.^1,2^ SBME can also be useful in scenarios that require teamwork and algorithms, such as when using Advanced Cardiac Life Support (ACLS). By utilizing SBME in a way that optimizes the effective features of this training method, the goal of improving learners’ ability to provide high-quality care to patients experiencing cardiac emergencies can be achieved.

Rapid cycle deliberate practice (RCDP) is a form of simulation built on Ericsson's teachings regarding expert skill acquisition through deliberate practice with real-time feedback.^3^ This innovative approach uses mastery learning and repetition to ensure that participants gain knowledge through overlearning, perfection of microskills, and creation of muscle memory.^4^ The more traditional simulation model features a case scenario for learners to work through, followed by a debriefing session.^5^ Issenberg and colleagues have suggested that the features of SBME most associated with effective learning are provision of feedback to learners and repetitive practice.^6^ RCDP seeks to improve simulation by maximizing the amount of time allotted to these two features. Instructors can pause simulation scenarios when there is an opportunity for the team of learners to improve their performance on a task or to reinforce positive behaviors. Individualized feedback is provided, and the simulation case is either restarted from that point or rewound back to the beginning of the case, allowing learners the opportunity to implement things they have learned and to create muscle memory. Learners repeat actions and receive feedback until they consistently perform a skill correctly, at which point they can progress to the next stage of the scenario. The RCDP curriculum has been described as having three principles, which are psychological safety, expert feedback, and optimizing the number of correct repetitions.^1,4^ This method has been used to improve the performance and confidence of pediatric residents in resuscitation scenarios in several studies.^4,5,7,8^ Additionally, RCDP has been shown to improve pediatric residents’ abilities to perform intubation to a greater degree than did a traditional simulation curriculum.^9^

This curriculum represents a unique contribution to existing literature in *MedEdPORTAL* by providing an RCDP-based ACLS simulation curriculum for adult patients. Scenarios in this curriculum are designed to be delivered in person and include pulseless ventricular tachycardia, unstable supraventricular tachycardia, and unstable sinus bradycardia. Previous works in *MedEdPORTAL* have applied the RCDP method of simulation to similar scenarios with success, but these scenarios have only included cases for pediatric patient populations.^5,10,11^

We designed this curriculum for junior anesthesiology residents to address identified gaps related to ACLS and defibrillator use. It was first implemented as part of a larger boot camp curriculum aimed at preparing residents to care for patients in operative and perioperative areas. However, the curriculum can be generalized to patient care in any inpatient or procedural area with various types of staff members trained in ACLS.

## Methods

### Development

Time-sensitive interventions for acute cardiovascular disease can be effectively taught using repetitive training, such as RCDP-based SBME, that allows health care providers to respond instinctively. The simulation case files (Appendices A–C) contained three simulation cases that made use of RCDP. Each case took place in the setting of the postanesthesia care unit (PACU) and required learners to provide treatment for different cardiovascular emergencies using ACLS algorithms and effective teamwork and communication principles. As participants completed certain tasks, the simulation was paused to allow instructors to provide both praise and correction. The cases allowed learners to reach competency in critical functions of care, including assessing patient vital signs, identifying abnormal rhythms, and providing the appropriate care based on findings.

### Equipment/Environment

We recreated a simulated PACU within the simulation center. Each of the case environments was prepared with equipment required for a code situation, including a resuscitation cart, a defibrillator with pads, IV pumps, and other equipment available to learners upon request. A complete description of mannequins and other materials used can be found within the simulation case files (Appendices A–C). Lab images for Appendices A and B can be found in Appendices D and E, respectively.

### Personnel

Personnel requirements for this curriculum included physician and nonphysician instructors who received training on how to lead simulation-based activities and provide effective debriefing. All facilitators were members of the anesthesiology department at the implementing institution and received similar simulation instructor training. This instructor training included techniques used for simulation design and debriefing, as well as providing an understanding of simulation methodology. In addition to experience and training on traditional immersive simulation, instructors also received specialized simulation training on skills specific to the implementation of RCDP-based SBME, such as giving real-time, rapid feedback and guiding deliberate practice and overlearning through facilitation of multiple rounds of the same simulation. An attending anesthesiologist served as the content expert. Simulation operations staff were present to operate the mannequin. The role of embedded nurse was played by a single simulation educator. A patient history and case stem were provided to the group of learners as the simulation exercise began. Each group of learners was composed of five to six anesthesia residents. Learners were asked to designate a team leader to assign roles to each team member. These roles were rotated throughout the session.

### Implementation

This curriculum was implemented in a way that required learners to progress through scenarios with increasing difficulty. After a 15-minute prebrief and orientation to the simulated environment, participants were given the case stem and asked to care for the patient. A single learner was directed to enter the simulated care environment, which contained an unresponsive patient experiencing a cardiac emergency. This learner needed to recognize the patient's condition and the cardiac rhythm present on the monitor and to respond by calling for help and a crash cart. Upon receiving the call for help, the remainder of the learners entered the room and assisted with care. Learners also needed to assign roles and treat the patient appropriately per ACLS guidelines.^12^

As detailed in the simulation case scenarios (Appendices A–C), facilitators regularly paused learners to offer feedback and coaching on microskills, first focusing on skills required for the initial assessment of the clinical scenario such as checking for patient responsiveness and presence of a pulse. The case was then repeatedly rewound back to the beginning until mastery of initial skills was achieved, at which time facilitators allowed learners to progress to more advanced skills, such as operating the defibrillator. Learners were required to perfect each skill and repeatedly perform it perfectly throughout the simulation before moving on to the next. Stopping points were not predetermined but were determined by the actions or inactions of the learner group. Each simulation case scenario can be found in full in Appendices A–C. A list of critical actions for each of the cases can be found within the flow chart located in each scenario case. After each case was complete and mastery of microskills achieved, a short group debriefing was held using the structure found in Appendix F. One hour of time was dedicated to each case and debriefing.

### Debriefing

In these RCDP simulations, debriefing was provided by pausing the case based on team performance of tasks found in the actions checklists in Appendices A–C. Instructors utilized pauses during the activity to show learners opportunities for improvement or to reinforce desirable behaviors. When the learners mastered a skill, the case was started again by picking up immediately after the pause or, if more deliberate practice was needed, rewound to the beginning or a specific period of the case, depending on learner needs. In this curriculum, debriefing was also provided at the end of the activity. Learners were asked to give their reaction to the activity and to summarize what they had learned. Facilitators also used this time to reinforce key points related to clinical treatment, teamwork, and communication aspects of each case. A complete description of the debriefing plan can be found in Appendix F.

### Assessment

Assessment of team performance during the simulation activity was done by facilitator observations based on the checklists found in Appendices A–C. Learner actions and inactions were used to assess team competency during the simulation activity and to determine the need for additional instruction. Learners did not receive a score for their performance during this activity but were asked to self-evaluate certain aspects of the training, including team communication.

## Results

Since its development, we have used this RCDP curriculum for ACLS skills to teach a total of 84 individuals during eight offerings of the course. Participants were all junior anesthesiology residents at a single institution. Most learners expressed high levels of satisfaction with the curriculum's ability to meet educational objectives and improve their performance in a clinical setting.

Facilitators noted that by the end of the simulations, residents were communicating more effectively, working more cohesively as a team, and appropriately delegating roles. Additionally, residents seemed more confident in assuming a leadership role and applying ACLS algorithms. Finally, residents were able to successfully utilize the defibrillator in manual mode to defibrillate, pace, and synchronized cardiovert the simulated patients, as appropriate.

To evaluate the impact of this resource, we asked participants to provide qualitative comments to represent their reaction to this experience. Twenty-one of the participants were also asked to complete a survey requiring them to rank their level of agreement with several statements regarding the quality of their experience. Ninety-five percent of respondents strongly agreed that this activity would improve their performance in a clinical setting, 86% strongly agreed that the educational objectives had been met, and 100% agreed that the feedback they received was valuable.

The RCDP method appears to have been well received given that the qualitative comments provided by learners were very positive. In general, participants found the opportunity to get hands-on experience accompanied by frequent feedback to be very useful. Specific comments from the eight offerings of this simulation at our institution included the following:

• “Well done.”
• “Best sim session yet.”
• “Very valuable.”
• “Feeling more comfortable with leading codes feeling more comfortable with the AED [automated external defibrillator].”

Several participants provided constructive criticisms after their experience. These criticisms included comments asking for more explanation of RCDP. Many learners also asked that more SBME be included in their education.

## Discussion

By creating this curriculum, we have provided an SBME experience that can be used to prepare resident physicians for situations requiring application of ACLS. The curriculum is unique because it uses the RCDP model for simulation, which seeks to improve medical education by providing trainees with immediate feedback and multiple attempts to do things the right way. Another unique feature of the curriculum is its use of RCDP with adult simulated patients.

The curriculum was developed using American Heart Association ACLS guidelines to provide both learning objectives and RCDP checkpoints for learners. Curriculum development included a pilot phase involving the same level, type, and number of learners. A discovery during this pilot was that learners wanted an opportunity to briefly discuss their reactions and synthesize findings, as well as to ask any remaining questions. As a result, we incorporated a post-RCDP debriefing at the end of each case in subsequent sessions. Additionally, we discovered that residents were very inquisitive about the treatment and defibrillation of a pulseless patient and often asked related questions during the unstable supraventricular tachycardia and bradycardia cases. We made the decision to adjust the order of the cases to allow for exposure to treatment of a pulseless patient earlier in the curriculum.

All faculty involved in the implementation of this project were members of the anesthesiology department at a single institution. In addition to experience with and training on traditional immersive simulation, instructors also received specialized simulation training on skills specific to the implementation of an RCDP-based SBME, such as giving real-time rapid feedback and guiding deliberate practice through facilitation of multiple rounds of the same simulation. As part of this training, instructors had the opportunity to facilitate RCDP simulations and receive expert feedback on their facilitation effectiveness. The lead facilitator in the RCDP sessions (Erin Blanchard) has facilitated dozens of RCDP simulations with diverse learner groups. Because our institution is accredited by the Society for Simulation in Healthcare, frequent evaluations of each of our simulation courses have been conducted. Changes have been made based on the feedback from both learners and faculty. We believe that the postsimulation survey results from learners indicate that the curriculum satisfies the need for an effective RCDP-based SBME curriculum for acute cardiovascular care in adult populations. We also believe that the curriculum can be used by other institutions to improve the preparation of their resident physicians.

Some limitations of this resource are that the curriculum has only been used at our institution for anesthesiology residents and that the sample size of learners this resource has been tested on is relatively small. Opportunities for future improvement would be inclusion of more members of the care team, such as pharmacy and nursing, in the RCDP simulations, thereby contributing to interprofessional interactions and larger sample sizes. We believe that the positive feedback from residents and the previous success attributed to the RCDP method indicate that this curriculum can be used effectively by other institutions. We also believe that this curriculum need not be limited to anesthesiology residents. Due to the generalized nature of the ACLS guidelines this resource has been developed from, the curriculum can be of use to all specialties that care for adult patients. Although qualitative, our learners’ feedback was overwhelmingly positive, and we hope that the curriculum's use by other institutions can build upon this success. We also hope to expand scholarship around this curriculum to include quantitative measures.

## Appendices


Unstable Bradycardia Sim Case.docxUnstable SVT Sim Case.docxVTach Sim Case.docxUnstable Bradycardia Images.docxUnstable SVT Images.docxDebriefing Form.docx
*All appendices are peer reviewed as integral parts of the Original Publication.*

## References

[1] Taras J, Everett T. Rapid cycle deliberate practice in medical education—a systematic review. Cureus. 2017;9(4):e1180. 10.7759/cureus.1180 28540142 PMC5441688

[2] Cook DA, Hatala R, Brydges R, et al. Technology-enhanced simulation for health professions education: a systematic review and meta-analysis. JAMA. 2011;306(9):978–988. 10.1001/jama.2011.1234 21900138

[3] Ericsson KA. Deliberate practice and acquisition of expert performance: a general overview. Acad Emerg Med. 2008;15(11):988–994. 10.1111/j.1553-2712.2008.00227.x 18778378

[4] Hunt EA, Duval-Arnould JM, Nelson-McMillan KL, et al. Pediatric resident resuscitation skills improve after “rapid cycle deliberate practice” training. Resuscitation. 2014;85(7):945–951. 10.1016/j.resuscitation.2014.02.025 24607871

[5] Patricia K, Arnold J, Lemke D. Rapid cycle deliberate practice: application to neonatal resuscitation. MedEdPORTAL. 2017;13:10534. 10.15766/mep_2374-8265.10534 30800736 PMC6342166

[6] Issenberg SB, McGaghie WC, Petrusa ER, Gordon DL, Scalese RJ. Features and uses of high-fidelity medical simulations that lead to effective learning: a BEME systematic review. Med Teach. 2005;27(1):10–28. 10.1080/01421590500046924 16147767

[7] Lemke DS, Fielder EK, Hsu DC, Doughty CB. Improved team performance during pediatric resuscitations after rapid cycle deliberate practice compared with traditional debriefing: a pilot study. Pediatr Emerg Care. 2019;35(7):480–486. 27741071 10.1097/PEC.0000000000000940

[8] Magee MJ, Farkouh-Karoleski C, Rosen TS. Improvement of immediate performance in neonatal resuscitation through rapid cycle deliberate practice training. J Grad Med Educ. 2018;10(2):192–197. 10.4300/JGME-D-17-00467.1 29686759 PMC5901799

[9] Gross IT, Abrahan DG, Kumar A, et al. Rapid cycle deliberate practice (RCDP) as a method to improve airway management skills—a randomized controlled simulation study. Cureus. 2019;11(9):e5546. 10.7759/cureus.5546 31523589 PMC6721918

[10] Doughty C, Welch-Horan T, Hsu D, et al. Rapid cycle deliberate practice pediatric simulation scenarios. MedEdPORTAL. 2015;11:10134. 10.15766/mep_2374-8265.10134

[11] Lemke DS. Rapid cycle deliberate practice for pediatric intern resuscitation skills. MedEdPORTAL. 2020;16:11020. 10.15766/mep_2374-8265.11020 33241116 PMC7678026

[12] Panchal AR, Bartos JA, Cabañas JG, et al; Adult Basic and Advanced Life Support Writing Group. Part 3: adult basic and advanced life support: 2020 American Heart Association guidelines for cardiopulmonary resuscitation and emergency cardiovascular care. Circulation. 2020;142(16)(suppl 2):S366–S468. 10.1161/CIR.0000000000000916 33081529

