# Supplementary material for: Rapid Cycle Deliberate Practice: Application to Adult Advanced Life Support
Source: MedEdPORTAL. 2022 Aug 23;18:11269. doi: 10.15766/mep_2374-8265.11269 (PMC9395559; doi:10.15766/mep_2374-8265.11269)
Supplement: Supplementary file 1 — Unstable Bradycardia Sim Case.docxUnstable SVT Sim Case.docxVTach Sim Case.docxUnstable Bradycardia Images.docxUnstable SVT Images.docxDebriefing Form.docx [file mep_2374-8265.11269-s001.zip › B. Unstable SVT Sim Case.docx]

| **Appendix B: Unstable SVT Sim Case**  **SIMULATION CASE TITLE:** Unstable SVT RCDP  **AUTHORS:** Erin Blanchard & Tekuila Carter  **LEARNER AUDIENCE:** CA1 Anesthesia Residents | |
| --- | --- |
| **PATIENT NAME:** John Doe  **PATIENT AGE:** 55 y/o  **CHIEF COMPLAINT:** Increasing heart rate  **PHYSICAL SETTING:** PACU | |
|  | |
| **Brief narrative description of case** | You are here due to a page that this patient in PACU needs help from you, the PACU resident, because of increasing HR after arriving to PACU 10 minutes ago post uneventful lap chole. |
| **Primary Learning Objectives** | *At the end of the session, learners should be able to…*  *1. Demonstrate effective teamwork and communication*  *2. Appropriately designate roles*  *3. Effectively operate a defibrillator in manual mode*  *4. Successfully lead a team during a cardiac emergency*  *5. Accurately apply Adult Cardiac Life Support algorithms to patient care* |
| **Learner Preparation or Prework** | 1. **Welcome/Introductions** 2. **Purpose:**  - Our purpose today is to improve performance of resuscitation skills and team communication. In other words, we want to help you become a highly functional team that is capable of providing high quality and effective life-saving resuscitation for your patient. We want you to leave with a functioning knowledge of ACLS algorithms and highly effective team performance behaviors.  1. **What is RCDP:**  - RCDP stands for rapid cycle deliberate practice. Simulation in which you may have participated in the past usually has the format of a simulation experience followed by a debriefing session. Rapid cycle deliberate practice is DIFFERENT. RCDP allows learners to **practice skills repetitively, receive brief interspersed feedback, and has been shown to improve individual performance of clinical skills^1^.** - We will embed multiple opportunities for specific feedback, coaching, and repetitive PERFECT practice. - **RCDP combines team-based training with repetitive practice and progressive difficulty, allowing opportunities for learners to “try again” in a manner similar to a sports team or a professional athlete engaged in activities designed to result in PERFECT performance.** - Learners will achieve predefined goals in each round before progressing to the next level or difficulty. - We will tell you the situation, objectives, and the clinical scenario prior to your entering the simulation. Please use any cognitive aids that you would have with you in the clinical setting. - We will PAUSE during the simulation to **provide specific feedback and coaching**. - The feedback of RCDP **targets specific technical and nontechnical skills as learning points** that occur within a round of a case^1^. - Each time we will attempt to orient you to where are re-starting as a team. There **will be some modeling and choreographing of key behaviors**. We are seeking to help your team meet specific performance metrics. - After the pause, four options are possible:   - Restart: start simulation at the beginning of the round   - Rewind: resume simulation at a specified period of time   - Resume: from where you left off   - Round up: when the objectives for the round are met, we will start the next round - Please be patient; **RCDP is an effective learning process**.  1. **Confidentiality and video release**  - We ask that you not repeat or speak about the scenario encountered during your simulation session so that we can preserve the integrity of our cases for future learners. Additionally, all of our simulation sessions are recorded for accreditation purposes and all learners are required to sign a video release form if they consent to such.  1. **About the manikin and simulation environment:**  - The manikin has pulses, breathes, blinks, and will display a cardiac rhythm when attached to a monitor. The defibrillator is live; please do a visual and verbal clear prior to discharging electricity for safety reasons. Please use any/all items within the crash cart and push IV medications as necessary.  1. **Basic Assumption:**  - We hold the basic assumption that everyone involved in simulation activities at UAB is intelligent, capable, cares about doing their best, and wants to improve^2^.  1. **Debrief**  - Once the simulation session has concluded, we will debrief as a group.  1. **Psychological safety**  - We believe that simulation based medical education should occur in a supportive environment that allows the psychological safety of learners. Sometimes simulation can impact us in unexpected ways. We acknowledge that we all come from various backgrounds and life experiences. If you need someone to talk to after a simulation session, please see your facilitator after the simulation session is over.  1. **Safety Phrase:**  - If you for some reason feel ill or need immediate help due to an emergency, please use the phrase "This is not a simulation." This phrase indicates a true emergency has occurred that requires attention.  1. **Sim and patient safety**  - Please check your pockets before leaving today’s session. It is important that supplies from the simulation center remain in the simulation center and do not inappropriately end up in patient care areas. All the supplies in our center are labeled “Not for Human Use: Education Only”. |

1. Taras J, Everett T. Rapid Cycle Deliberate Practice in Medical Education - a Systematic Review. *Cureus.* 2017;9(4):e1180.
2. Rudolph JW, Raemer DB, Simon R. Establishing a safe container for learning in simulation: the role of the presimulation briefing. *Journal of the Society for Simulation in Healthcare*. 2014;9(6):339-349.

Materials and Setup:

| ***Simulator / Scenario / Files*** | ***AV Considerations*** |
| --- | --- |
| *Simulator to use:3G*  *Patient to use in Computer John Smith*  *Scenario Title in Computer: RCDP w/ LifePak 20- synchronized cardioversion*  *Supporting Files, Documents, etc: Labs, Xray* | *Video Recording*: YES / NO  *Video Streaming:* YES / NO  *Other:*  ***Due to accreditation requirements, all simulation debriefings will be recorded.*** |

| ***Initial Simulator Setup*** | |
| --- | --- |
| *Clinical Setting* | PACU |
| *Bed Type* | Stretcher |
| *Body Position* | supine |
| *IV Access* | One working, either arm |
| *Wounds/Dressings* | 3 gauze and medipore tape (one at the umbilicus, one patient’s right upper quadrant of abdomen, one patient’s right lower quadrant of abdomen) all without any drainage |
| *Moulage* | No |
| *Wig* | No |
| *Arm Band* | Yes |

| ***Monitors*** | ***NOTES*** |
| --- | --- |
| *Heart Rate* | Yes |
| *NIBP* | Yes |
| *Arterial Line* | Available upon request |
| *CVP* | Available upon request (AFTER REQUESTING CENTRAL LINE INSERTION KIT) |
| *Respiratory Rate* | Yes |
| *Oxygen Saturation* | Yes |
| *End Tidal CO_2_* | Available upon request |
| *Temperature* | Available upon request |

| ***Equipment / Supplies*** | ***NOTES*** |
| --- | --- |
| CVL setup | Connected to working drain bag; hidden under bed; available upon request |
| Defibrillator | on top of crash cart |
| Fully stocked crash cart | Outside of room; MULTIPLE LOCKS |
| Normal Saline | Connected to patient’s IV. Not on pump |
| IV pump with 2 channels | On IV pole in room |
| Defibrillator pads | Adult; multiple extra sets in or available to put in crash cart |
| Stool | In room |
| Ambu bag | At head of bed |
| Nasal cannula | On patient at start of case |
| Pressure bag | In bottom drawer of crash cart |
| Extra bags of fluids | Need:   - Multiple 1L bags - Multiple 100ml or 50ml bags - 1 or 2 250-ml bags |

| ***Additional Setup/Environmental Notes:*** |
| --- |
|  |

| **Initial Presentation** | | | |
| --- | --- | --- | --- |
| **Initial vital signs** | HR: 178 bpm, BP: 72/50, O2 Sat: 95% Temp: 98.2 | | |
| **Overall Setting and Appearance** | You are here due to a page that this patient in the PACU needs help from you, the PACU resident, because of increasing HR after arriving to PACU 10 minutes ago post uneventful lap chole. | | |
| **Confederates (e.g., standardized participants) and their roles in the room at case start** | One of the instructors should portray the role of a PACU nurse. The remaining instructors will be in the room to provide feedback. | | |
| **HPI** | 55 y/o male with no past medical history status post laparoscopic cholecystectomy. Pt does not take any medications. | | |
| **Past Medical/Surgical History** | **Medications** | **Allergies** | **Family History** |
| Laparoscopic Cholecystectomy, No other significant history. | None | None | None |
| **Physical Examination** | | | |
| **General** | Within normal limits | | |
| **HEENT** | Within normal limits | | |
| **Neck** | Supple | | |
| **Lungs** | Clear to auscultation | | |
| **Cardiovascular** | Tachycardic | | |
| **Abdomen** | Absent bowel sounds | | |
| **Neurological** | Within normal limits | | |
| **Skin** | Within normal limits | | |
| **GU** | GU exam not applicable | | |
| **Psychiatric** | Within normal limits | | |

| **Instructor Notes - Changes and CASE Branch Points** |
| --- |

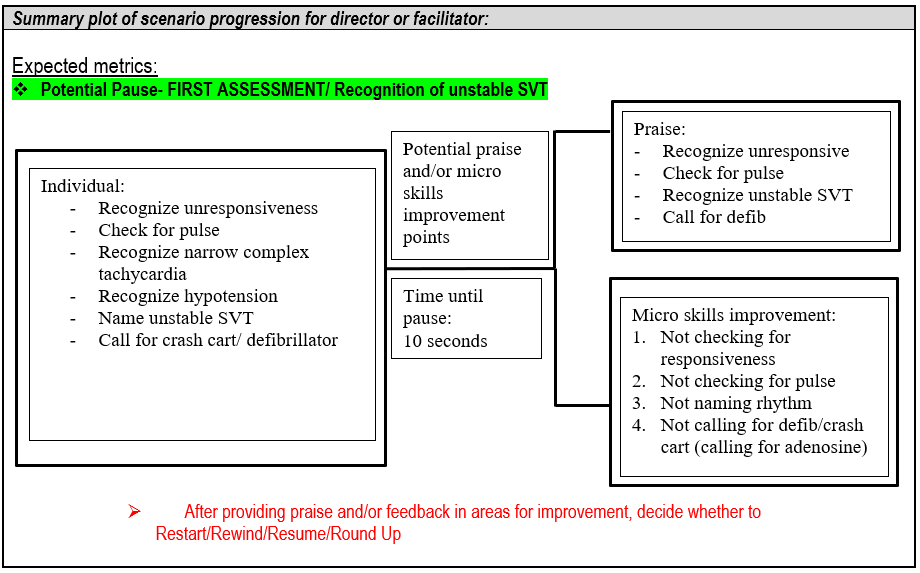


**
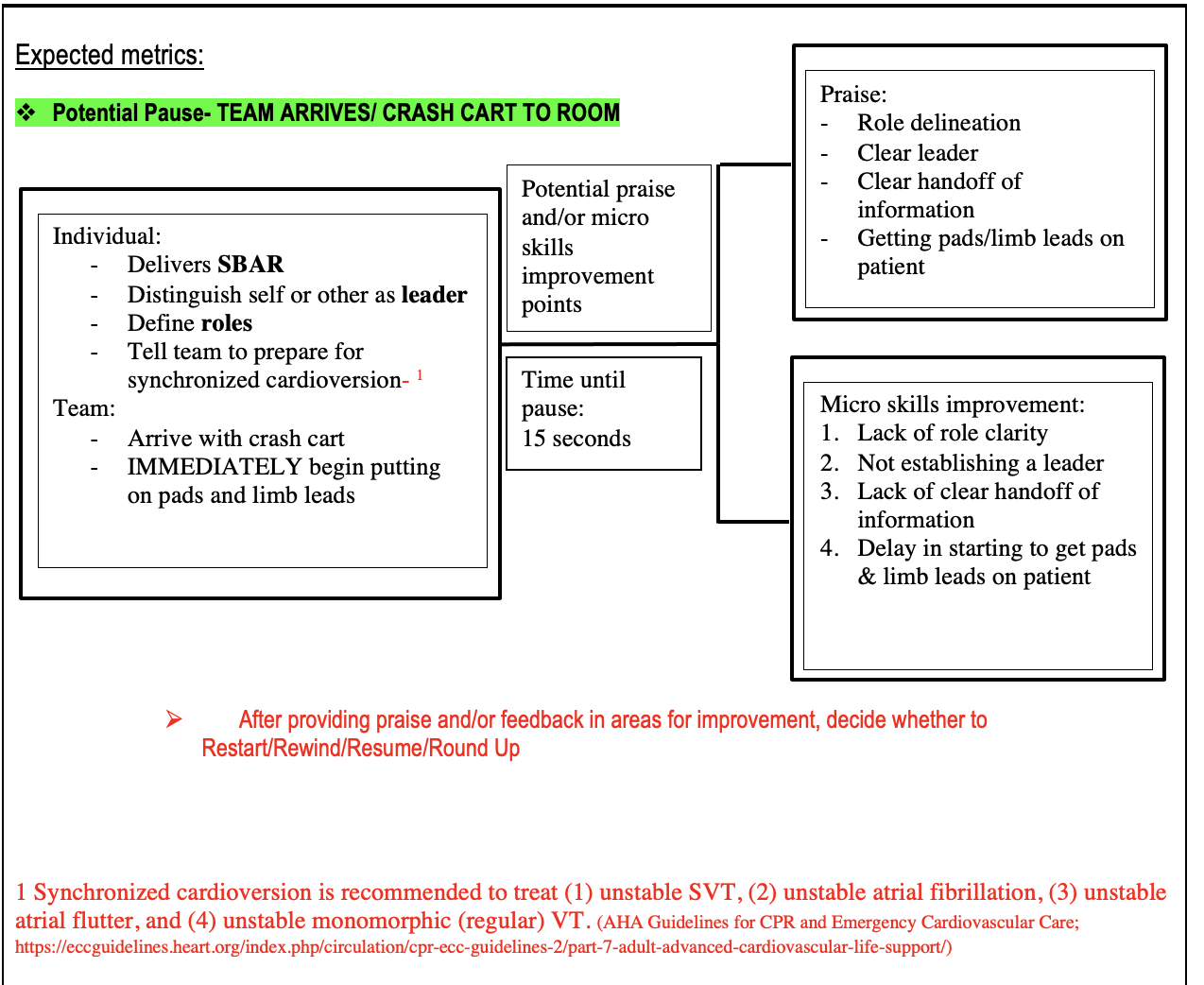
**

**
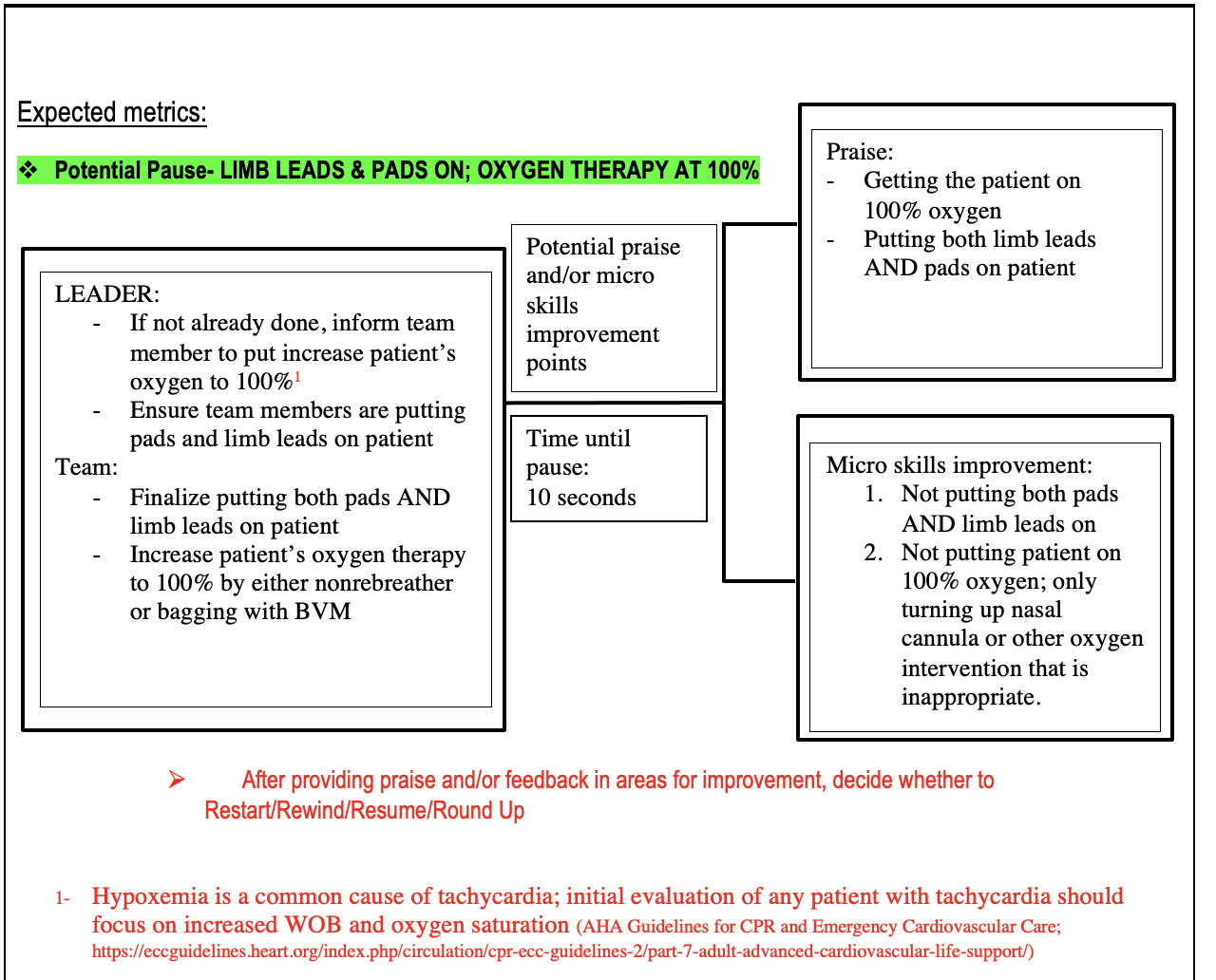
**


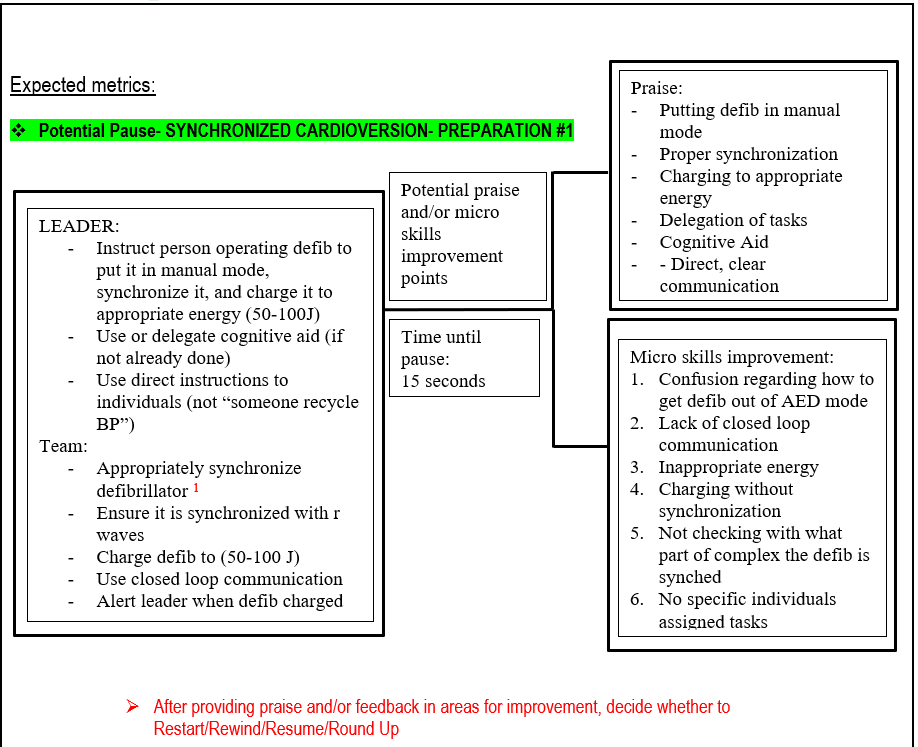


**
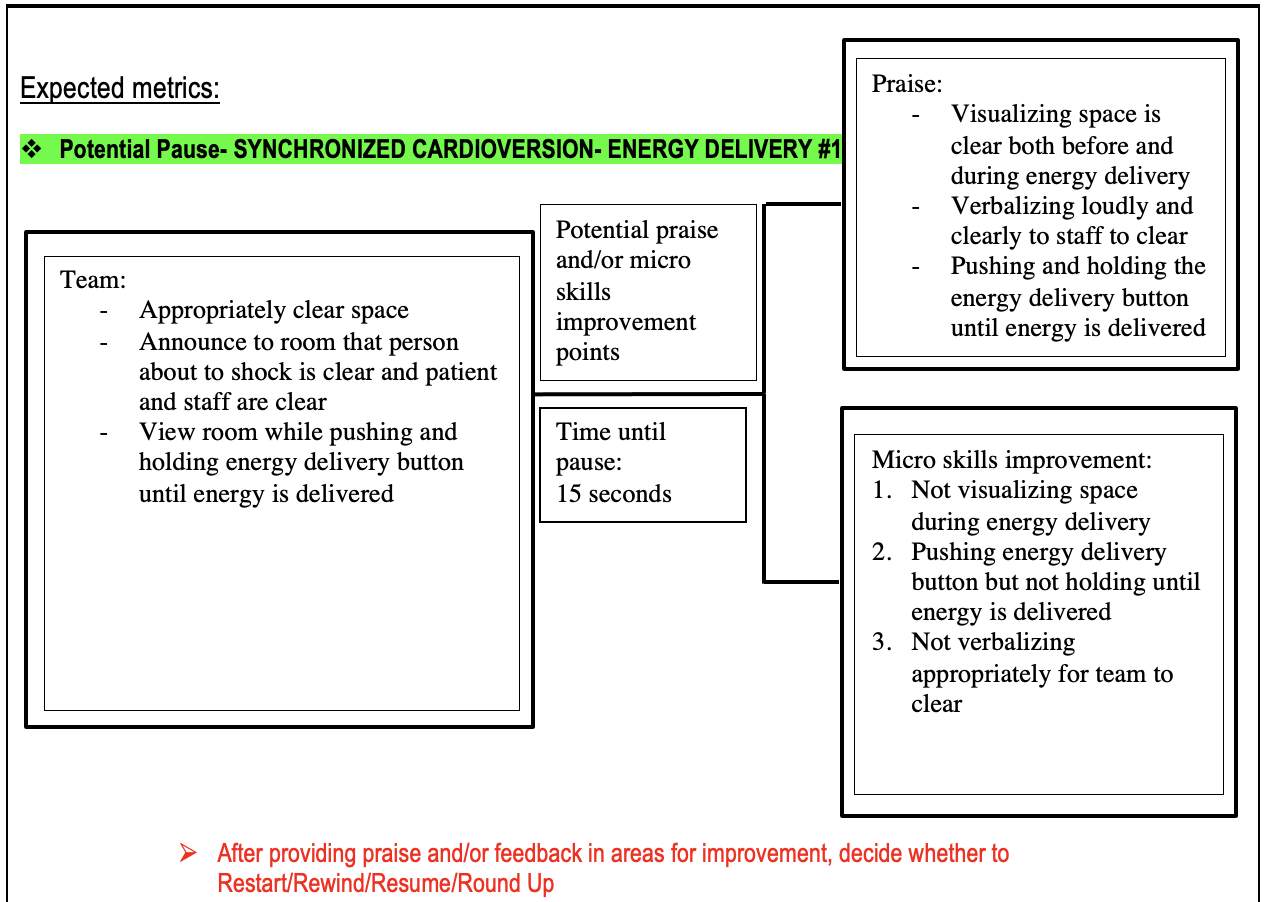
**

**Round 2:**

**
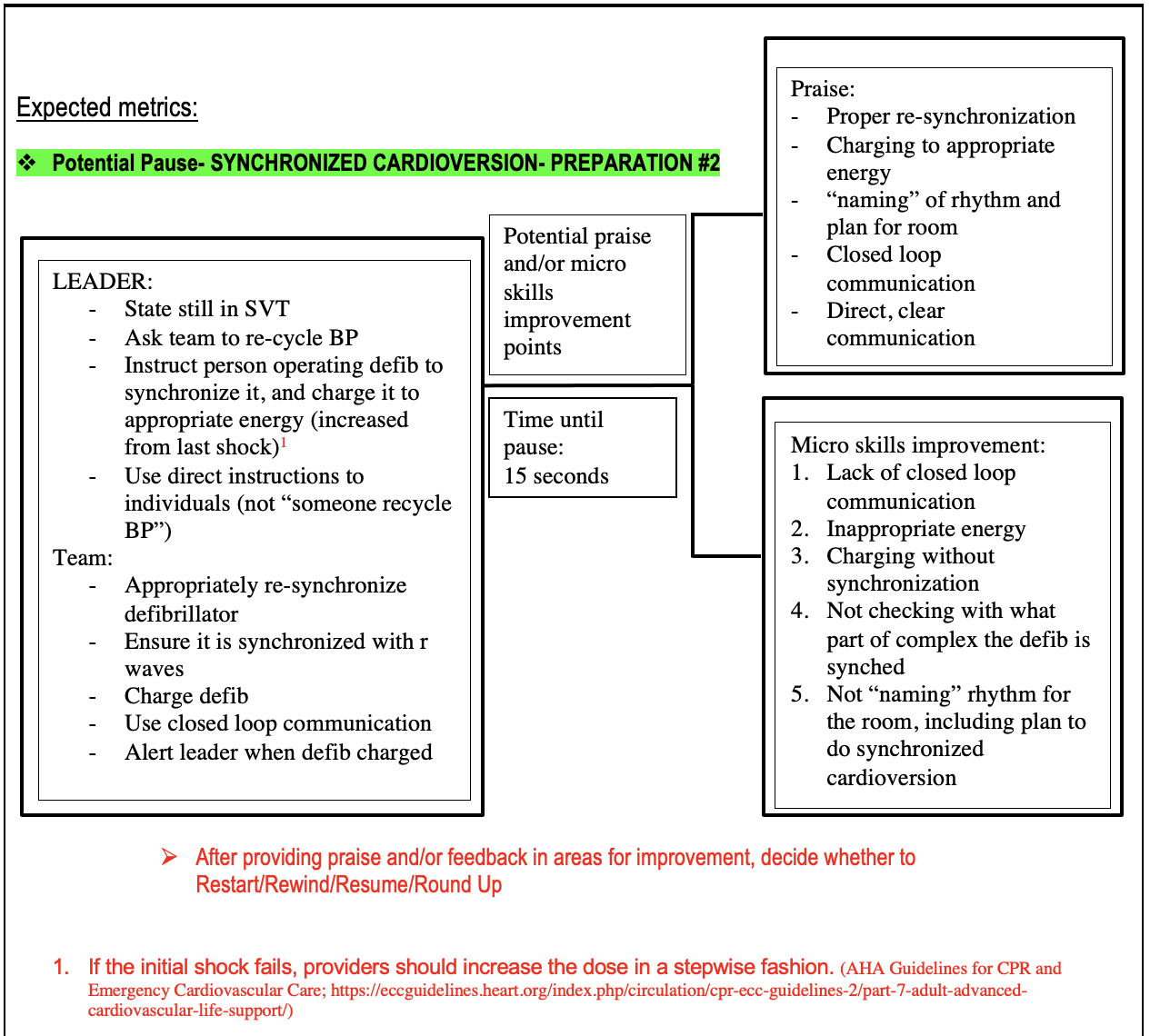
**


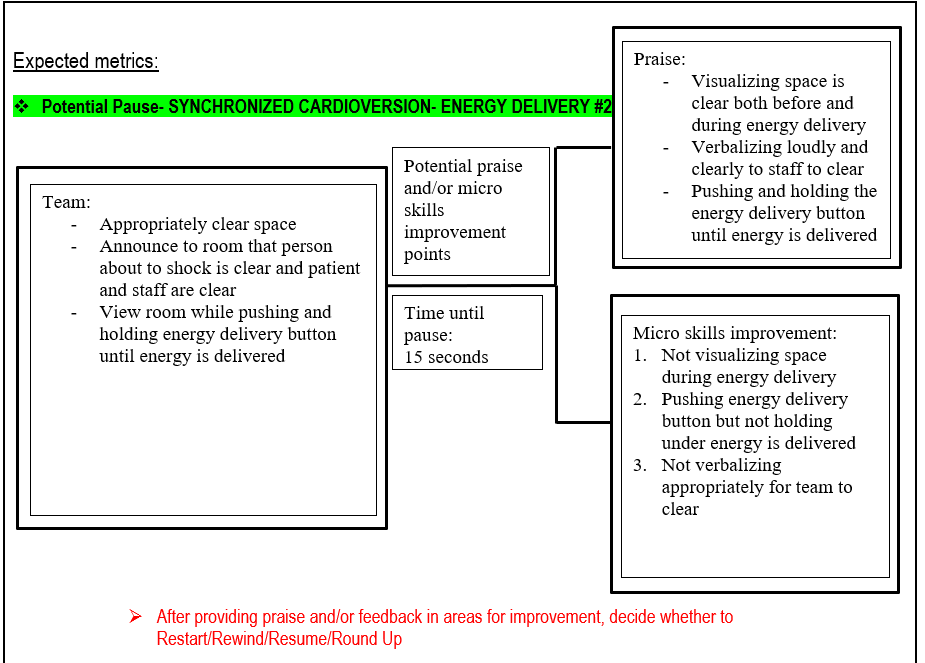


**Round 3:**


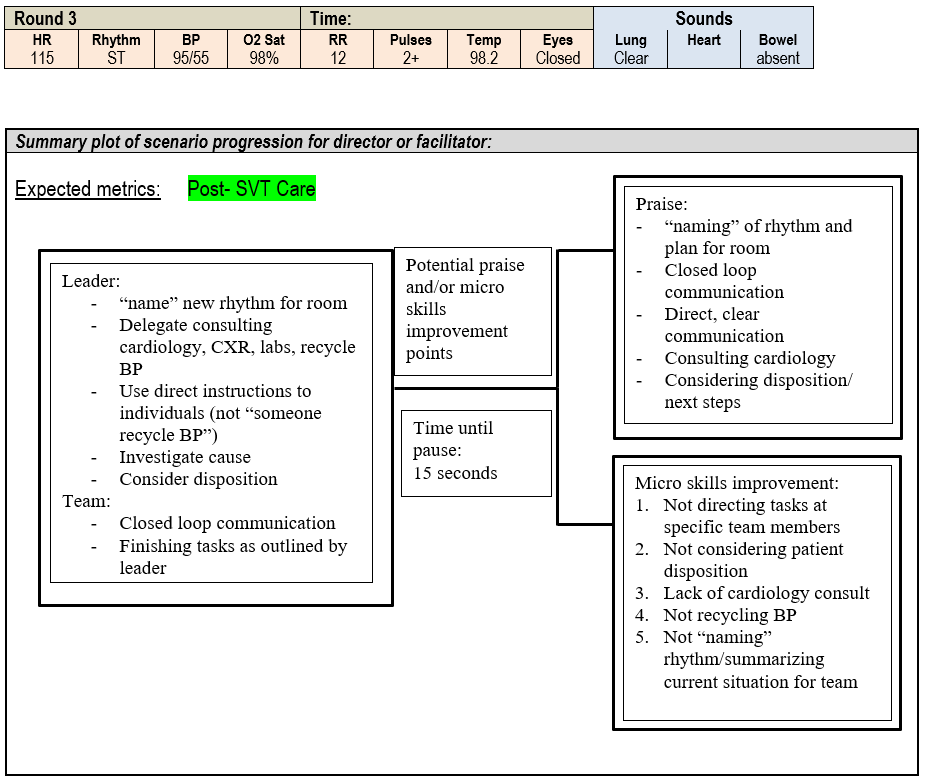


**Ideal Scenario Flow**

*One learner enters the room to assess the patient. After recognizing that patient is unresponsive and hypotensive the learner checks for a pulse and recognizes narrow complex tachycardia. The learner will name the rhythm unstable SVT and call for a crash cart and defibrillator. The rest of the team will arrive, and the original learner will deliver SBAR and be distinguished as the leader. The team immediately begins putting pads and limb leads on the patient. Other teammates will define their roles. The leader will then instruct the team to prepare for synchronized cardioversion. Patient’s oxygen increased to 100% by nonrebreather or bagging with BVM. The leader will instruct the person operating the defibrillator to put it in manual mode, synchronize it, and charge it to 50-100J. Leader will then instruct the team to appropriately clear from the patient and announce to the room that shock will be delivered. Shock is then delivered. The rhythm is immediately analyzed, and the leader will state that the patient is still in SVT. Leader will instruct a person to re-cycle blood pressure and the defibrillator operator will be instructed to synchronize it and charge it to appropriate energy. Once again, the leader will instruct all team member to “clear”, and the charge will be delivered. The new rhythm is analyzed and named sinus tachycardia by the team leader. The team will then delegate consulting and consider disposition.*

**Anticipated Management Mistakes**

1. *Not evaluating patient for responsiveness and pulse: The first learner to enter the room must assess the hypotensive patient for responsiveness and pulse. Failure to do so will require a pause of the case to allow for coaching of the learner. The case will then start from the beginning.*
2. *Failure to define roles and use closed loop communication: Clearly defined roles and closed loop communication will be assessed by instructors. Failure by learners to display these behaviors will lead to a pause in the simulation for micro skill improvement.*
3. *Inappropriate use of adenosine: Learners should understand that Adenosine may only be used in management of stable SVT and is not appropriate for treating unstable SVT*.
4. *Failure to use defibrillator in manual mode: Learners that have not used a defibrillator may require instruction in this area.*
5. *Not placing leads on patient properly: Learners should receive instruction in this area if instructors feel the necessity.*
