# Supplementary material for: Rapid Cycle Deliberate Practice: Application to Adult Advanced Life Support
Source: MedEdPORTAL. 2022 Aug 23;18:11269. doi: 10.15766/mep_2374-8265.11269 (PMC9395559; doi:10.15766/mep_2374-8265.11269)
Supplement: Supplementary file 1 — Unstable Bradycardia Sim Case.docxUnstable SVT Sim Case.docxVTach Sim Case.docxUnstable Bradycardia Images.docxUnstable SVT Images.docxDebriefing Form.docx [file mep_2374-8265.11269-s001.zip › D. Unstable Bradycardia Images.docx]

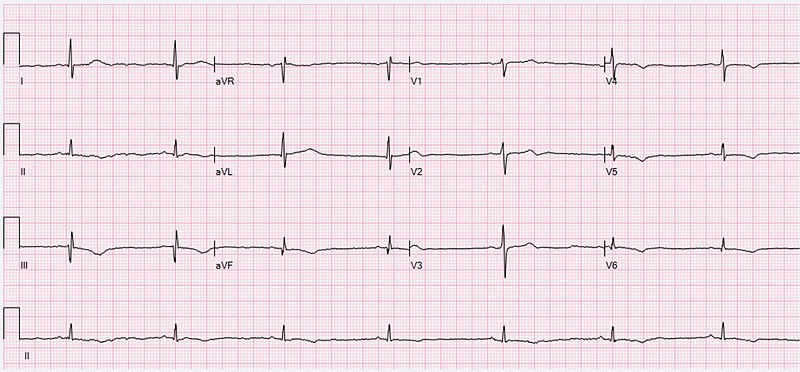


Image by Ewingdo, retrieved from https://commons.wikimedia.org/wiki/File:ECG_Sinus_Bradycardia_43_bpm.jpg on 9/16/21. Creative Commons License associated: https://creativecommons.org/licenses/by-sa/4.0/deed.en
