# Supplementary material for: Rapid Cycle Deliberate Practice: Application to Adult Advanced Life Support
Source: MedEdPORTAL. 2022 Aug 23;18:11269. doi: 10.15766/mep_2374-8265.11269 (PMC9395559; doi:10.15766/mep_2374-8265.11269)
Supplement: Supplementary file 1 — Unstable Bradycardia Sim Case.docxUnstable SVT Sim Case.docxVTach Sim Case.docxUnstable Bradycardia Images.docxUnstable SVT Images.docxDebriefing Form.docx [file mep_2374-8265.11269-s001.zip › F. Debriefing Form.docx]

| ***REACTIONS:*** *The purpose of this section is to clear the air so a learning conversation can occur. Try to tie reactions to learning objectives you plan to cover. Try to frame in emotions. NOT WHAT DID YOU THINK,* ***How do you feel****?* |
| --- |
| In one or two words, tell me how you’re feeling right now. |

| **Plus-Delta** | |
| --- | --- |
| **Individual & Team Treatment of Pathology**  *Key debriefing points:*   \| *Vtach/Defibrillation** \| *SVT/Synchronized Cardioversion** \| *Bradycardia/Pacing** \| \| --- \| --- \| --- \| \| - *Time from recognition to initial compressions* - *Backboard* - *Rate/Depth of compressions* - *Rhythm analysis* - *Defibrillator use* - *Epi- assembly, use, timing, dose* - *Seeking cause of event* - *Use of rounds of CPR* \| - *Rhythm analysis* - *Stable vs Unstable* - *Adenosine- use, timing, dose* - *Defibrillator use* - *Seeking cause of event* \| - *Rhythm analysis* - *Stable vs Unstable* - *Atropine- use, timing, dose* - *Defibrillator use* - *Seeking cause of event* \| | |
| What do you feel went well regarding your individual, and the team as a whole’s, treatment of *insert pathology (unstable bradycardia, supraventricular tachycardia, VTach)*? | What do you feel could have been improved regarding your individual, and the team as a whole’s, treatment of *insert pathology (unstable bradycardia, supraventricular tachycardia, VTach)*? |
|  |  |
| **Teamwork and Communication**  *Key debriefing points:*   \| *Vtach/Defibrillation* \| *SVT/Synchronized Cardioversion* \| *Bradycardia/Pacing* \| \| --- \| --- \| --- \| \| - *Calling for help* - *Establishing a leader*** - *“Naming the rhythm”* - *Role delegation* - *Closed-loop communication* - *Clearing before shock* \| - *Calling for help* - *Establishing a leader* - *“Naming the rhythm”* - *Role delegation* - *Closed-loop communication* - *Clearing before shock* \| - *Calling for help* - *Establishing a leader* - *“Naming the rhythm”* - *Role delegation* - *Closed-loop communication* \| | |
| What do you feel went well regarding your teamwork and communication? | What do you feel could have been improved regarding your teamwork and communication? |

| ***SUMMARY:*** *Ask learners to summarize their “take-aways.”* |
| --- |
| What are your take-aways from that experience? Do you have any final questions or concerns related to caring for a patient with *insert pathology (unstable bradycardia, supraventricular tachycardia, VTach)*? |

**It may be helpful to reference the latest applicable American Heart Association algorithm during discussion.*

***May look different for different types of learners. Refrain from commenting on certain personality types to maintain psychological safety.*
